# Microvascular responses in association with recent and chronic exposure to particulate air pollution in school children

**DOI:** 10.1186/2049-3258-73-S1-P18

**Published:** 2015-09-17

**Authors:** Eline Provost, Nelly Saenen, Michal Kicinski, Tijs Louwies, Karen Vrijens, Luc Int Panis, Patrick De Boever, Tim Nawrot

**Affiliations:** 1 Hasselt University, Diepenbeek, Belgium; 2 Centre for Environmental Sciences, Hasselt University, Diepenbeek, Belgium; 3 Flemish Institute for Technological Research (VITO), Mol, Belgium

## Background and aims

Microvascular changes may represent an underlying mechanism through which particulate matter (PM) exposure contributes to cardiovascular disease development. We investigated the effect of both recent and chronic exposure to PM on the microcirculation, exemplified by retinal vessel calibers, in a panel of healthy children.

## Methods

225 children (49% girls; mean age 9.9 years) were recruited at two primary schools in Belgium. Participating children were examined three times at school over the course of the school year, during which the fundus of both eyes was photographed. The caliber of the retinal blood vessels was summarized as the central retinal arteriolar/venular equivalent (CRAE/CRVE). Recent exposure to PM_2.5_ was measured at the school prior to the examination. Residential proximity to major roads was used as a proxy for chronic exposure. The effect of recent and chronic exposure to PM on retinal vessel caliber was estimated using mixed models, while adjusting for gender, age, BMI, blood pressure, heart rate, birth weight, time and day of examination, mother's education and passive smoking.

## Results

Each doubling in recent exposure to PM_2.5_ was associated with a 0.51 (95% CI: 0.17 to 0.86; p=0.0035) narrowing of the retinal arteriolar caliber (CRAE), while venular caliber (CRVE) widened 0.55 (95% CI: 0.06 to 1.04; p=0.029). Children living twice as close to a major road had 0.84 (95% CI: -0.0044 to 1.68; p=0.051) narrower arterioles.

## Conclusions

We show that vessel calibers of the retinal microcirculation of healthy children aged 8 to 12 years respond to recent PM exposure. Additionally, children living closer to major roads showed smaller calibers of their arterioles. Since changes in the microcirculation have been associated with cardiovascular disease development, these results suggest that the microcirculation is a pathophysiological target for air pollution from a young age onwards.

